# Spoon-Shaped Indentations in the Skulls of the New-Born

**Published:** 1901-01-26

**Authors:** 


### SPOON-SHAPED INDENTATIONS IN THE SKULLS OF THE NEW-BORN.

Many of us have seen these spoon-shaped indentations on the skulls of new-born children, and no doubt some of us have observed that in certain cases they have in course of time filled up and been forgotten. This, however, is a fortunate ending that does not always follow. Dr. Munro Kerr[1] says that indentations of the fœtal skull may be either spoon- or furrow-shaped. The furrow-shaped depression is much the less serious, and seldom gives rise to much immediate trouble. The spoon-shaped indentations, however, although in the majority of cases they disappear and leave no ill-effects, may remain as a permanent deformity, and may give rise to such marked disturbances as to end in death. As to cause, it seems probable that while the furrow-shaped depressions may in some cases be due to forceps, the spoon-shaped ones are caused in most cases by the head being pressed against an unduly projecting sacral promontory, or occasionally against an undue prominence of the ileo-pectineal eminence. Many dodges have been tried, generally unsuccessfully, with the object of elevating the depressed bone. But Dr. Kerr has discovered a very interesting thing. He says that the bones of the fœtal skull are very resilient, and that consequently a very little force applied to the depression from the inside, if applied early, is sufficient to remove the indentation. But how to apply the force? That is the question. Dr. Kerr hopes that he has found the answer. He had a case in which this deformity was met with, and while looking at the depression it occurred to him that by firm compression of the head antero-posteriorly sufficient pressure might be exerted on the depressed bone to make it spring out. He hardly expected so simple a manœuvre to be so successful, "but on the first attempt the depression came out, producing a sound as when a dent in a felt hat is removed." After this success he tried the effect on artificially produced depressions in still-born infants, and in all cases found such indentations to be removed by antero-posterior compression, indentations of the parietal bones especially coming out with great ease. Two other cases are added in which the same treatment was successful. This certainly is a thing to remember, and to try.

[1] Brit. Med. Jour., Jan. 19.

